# The Crosstalk Between Saliva Bacteria and Fungi in Early Childhood Caries

**DOI:** 10.3389/fcimb.2022.845738

**Published:** 2022-02-14

**Authors:** Ye Tu, Zhiyan Zhou, Chang Shu, Yuan Zhou, Xuedong Zhou

**Affiliations:** ^1^ State Key Laboratory of Oral Diseases & National Clinical Research Center for Oral Diseases, West China Hospital of Stomatology, Sichuan University, Chengdu, China; ^2^ Department of Cariology and Endodontics, West China Hospital of Stomatology, Sichuan University, Chengdu, China; ^3^ Department of Periodontology, Peking University School and Hospital of Stomatology, Peking University, Beijing, China; ^4^ Department of Pediatric Dentistry, West China Hospital of Stomatology, Sichuan University, Chengdu, China

**Keywords:** microbiome, mycobiome, saliva, early childhood caries, dental caries

## Abstract

Early childhood caries (ECC) is the most prevalent oral disease in children, which greatly affects the quality of life and health condition of the patients. Although co-infection of oral streptococci and fungi has been well recognized in the development of ECC, the correlation between other core members of oral mycobiome and ECC progression remains unclear. In the current study, saliva samples obtained from severe ECC (SECC), ECC, and caries-free children were collected, and both V3–V4 16S rRNA and ITS1 rRNA gene amplicon sequencing were performed to investigate the salivary bacterial and fungal profiles. Significant alteration of salivary fungal community in SECC/ECC children was observed compared with the caries-free control. The typing analysis determined the fungal community into five fungal types, which influenced the structure of salivary bacteria. By performing Spearman correlation analysis, carious phenotypes were positively related to *Fusobacterium* but negatively linked to *Neocosmospora*, and a significant correlation of cross-kingdom taxonomic pairs was identified. Our work demonstrated the interactions between oral bacteria and fungi at the community level, which may advance our knowledge on the etiological role of bacteria/fungi in the development of ECC and promote better management of this disease.

## Introduction

Early childhood caries (ECC) refers to caries occurring on deciduous dentition and is identified as one or more decayed (d), missing (m), or filled (f) surface or tooth in the primary tooth in children at 71 months of age or younger (Lis and Kuramitsu, 1997; Anil and Anand, 2017; Pierce et al., 2019). Severe ECC (SECC) is manifested as one or more cavitated, filled, or missing (due to caries) smooth surfaces in primary maxillary anterior teeth, or dmfs score ≥4 (age 3 years), ≥5 (age 4 years), or ≥6 (age 5 years), with younger onset age and higher morbidity (Drury et al., 1999). Being the most common chronic disease in children, ECC afflicts many population groups with prevalence rates ranging from 23% to 98% all over the world (Hu et al., 2011; Dye et al., 2015; Castillo et al., 2019; Pierce et al., 2019), causing heavy financial and medical burdens to the family and society (Rashewsky et al., 2012).

The etiology of ECC has been largely related to polymicrobial infection, accompanied by environmental, maternal, behavioral, and socioeconomic factors (Kim Seow, 2012; Fontana, 2015; Hemadi et al., 2017; Xiao et al., 2018). The involvement of *Streptococcus mutans* in ECC has been well recognized, largely accredited to its acidogenicity, acidurity, and capability of producing polysaccharides (Hajishengallis et al., 2017; Hemadi et al., 2017; Momeni et al., 2020). *Candida albicans* has also been demonstrated to be involved in ECC development, and its synergistic interaction with *S. mutans* greatly enhances the virulence of the biofilm (Falsetta et al., 2014; Hwang et al., 2017; Ellepola et al., 2019). Other than the extensively investigated *C. albicans*, the complicated component of oral mycobiome and the biofilm formation capacity of several fungal taxa have been discovered (Ghannoum et al., 2010; Angiolella et al., 2020; Chakraborty et al., 2020), suggesting the potential impact of fungal taxa on oral health. Furthermore, the interaction between microbiome and mycobiome has been observed in certain diseases (Hoarau et al., 2016; Azzam et al., 2020), indicating the profound influence of polymicrobial dysbiosis in disease development. However, the role of oral fungal community in ECC progression and the cross-kingdom interaction between oral bacteria and fungi still need further investigation.

The clinical validity and convenience of saliva test have been well suggested (Zhang et al., 2016; Kaczor-Urbanowicz et al., 2019; Fernandes et al., 2020), while the dental explorer which is used for plaque collecting may cause fear and anxiety in children (Leal et al., 2013), leaving a more preferable test method of saliva to kids. So far, the only few published studies involving whole oral mycobiome and ECC were either focusing solely on a fungal community without referring to oral bacteria (Fechney et al., 2019; Cui et al., 2021) or using dental plaque or oral swab samples (de Jesus et al., 2020; de Jesus et al., 2021). Hence, in the current study, we intended to 1) capture the saliva microbial (bacterial and fungal) community variation in different oral health status, 2) identify potential microbial biomarkers of healthy and ECC status, and 3) uncover the interaction between the oral microbiome and mycobiome in caries development. Results here illustrated the alteration of saliva microecosystem as caries progressed with certain taxa significantly enriched in caries children, and the potential interaction between saliva bacterial and fungal community was observed.

## Methods

### Study Population, Oral Examination, and Sample Collection

The current study was approved by the Institutional Review Board of West China Hospital of Stomatology (WCHSIRB-D-2017-031). Children under 72 months of age were recruited except for those who met the following exclusion criteria: antibiotic or fluoride treatment within 3 months, mixed dentition, systemic diseases, and acute infection. The comprehensive oral examination was conducted by two professional dentists according to the WHO Basic Oral Health Survey Methods 1997 with good consistency (intra-examiner Kappa value = 0.90, inter-examiner Kappa value = 0.90). The number of carious teeth and surfaces was assessed with index variables of decayed, missing due to decay, or filled (dmfs; dmft). The diagnosis of ECC and SECC followed the instructions as previously described (Drury et al., 1999). Five milliliters of whole non-stimulated saliva was collected using the spitting method as described previously with minor modification (Navazesh, 1993). Briefly, samples were collected between 9:00 and 11:00 a.m., and participants were refrained from toothbrushing, eating, or drinking at least 2 h prior to sample collection. Participants were instructed to rest for 5 min with minimum orofacial movements and were then requested to slightly tilt the head forward to accumulate saliva in the floor of the mouth. Saliva was spitted into 50-ml centrifuge tubes every 1 min, and approximately 10 min was consumed collecting 5 ml non-stimulated saliva from children. All samples were immediately placed on dry ice and transported to a −80°C freezer for storage prior to further analysis.

### DNA Extraction, Amplification, and Illumina MiSeq Sequencing

Total DNA was extracted using the TIANamp bacteria DNA Kit (Tiangen, Beijing, China), according to the protocol of the manufacturer. DNA extraction was checked on 1% agarose gel, and DNA concentration and purity were determined with NanoDrop 2000 UV–vis spectrophotometer (Thermo Scientific, Wilmington, USA). The bacterial 16S rDNA V3–V4 region and fungal ITS-1 region were amplified using primer pairs 338F (5′-ACTCCTACGGGAGGCAGCAG-3′) and 806R (5′-GGACTACHVGGGTWTCTAAT-3′) for bacteria, or ITS1F (5′-CTTGGTCATTTAGAGGAAGTAA-3′) and ITS2R (5′-GCTGCGTTCTTCATCGATGC-3′) by an ABI GeneAmp^®^ 9700 PCR thermocycler (ABI, CA, USA). The PCR amplification of 16S rRNA and ITS gene was performed as follows: initial denaturation at 95°C for 3 min, followed by 27 cycles of denaturing at 95°C for 30 s, annealing at 55°C for 30 s and extension at 72°C for 45 s, and single extension at 72°C for 10 min, and end at 4°C. The PCR mixtures contain 5× TransStart FastPfu buffer 4 μl, 2.5 mM dNTPs 2 μl, forward primer (5 μM) 0.8 μl, reverse primer (5 μM) 0.8 μl, TransStart FastPfu DNA Polymerase 0.4 μl, BSA 0.2 μl, template DNA 10 ng, and finally ddH_2_O up to 20 μl. PCR reactions were performed in triplicate. The PCR product was extracted from 2% agarose gel and purified using the AxyPrep DNA Gel Extraction Kit (Axygen Biosciences, Union City, CA, USA) according to the instructions of the manufacturer and quantified using Quantus™ Fluorometer (Promega, USA). Purified amplicons were pooled in equimolar and paired-end sequenced on an Illumina MiSeq PE300 platform/NovaSeq PE250 platform (Illumina, San Diego, USA) according to the standard protocols by Majorbio Bio-Pharm Technology Co., Ltd. (Shanghai, China). The raw reads were deposited into the NCBI Sequence Read Archive (SRA) database (https://www.ncbi.nlm.nih.gov/bioproject/PRJNA790078, https://www.ncbi.nlm.nih.gov/bioproject/PRJNA790007).

### Processing of Sequencing Data

The raw 16S and ITS1 rRNA gene sequencing reads were demultiplexed and quality-filtered by fastp version 0.20.0 (Chen et al., 2018) and merged by FLASH version 1.2.7 (Magoč and Salzberg, 2011) with the following criteria: i) the 300-bp reads were truncated at any site receiving an average quality score of <20 over a 50-bp sliding window, the truncated reads shorter than 50 bp were discarded, and reads containing ambiguous characters were also discarded; ii) only overlapping sequences longer than 10 bp were assembled according to their overlapped sequence. The maximum mismatch ratio of the overlap region is 0.2. Reads that could not be assembled were discarded; iii) samples were distinguished according to the barcode and primers, and the sequence direction was adjusted, exact barcode matching, two nucleotide mismatches in primer matching.

Operational taxonomic units (OTUs) with 99% similarity were clustered using UPARSE version 7.1 (Edgar, 2013), and chimeric sequences were identified and removed. The taxonomy of each OTU representative sequence was analyzed by RDP Classifier version 2.2 (Wang et al., 2007) against the bacterial 16S rRNA database (SILVA 138) and fungal ITS database (UNITE 8.0) under the confidence threshold of 0.7.

### Bioinformatics and Statistical Analysis

Before bioinformatic analysis, sequencing reads of all samples were standardized by rarefying OTU tables to the minimum number of reads. Analyses were performed by using the online platform of Majorbio Cloud Platform (www.majorbio.com). Kruskal–Wallis *H* test and Wilcoxon rank-sum test were used to compare the differences in taxa between three groups or two groups. Alpha-diversity was calculated in terms of Shannon and Chao indexes and was compared by Wilcoxon rank-sum test. Beta-diversity was assessed by principal coordinates analysis (PCoA) using Bray–Curtis distance and permutational multivariate analysis of variance (PERMANOVA) with a permutation of 999. Analysis of similarities (ANOSIM) values were constructed in R (version 3.3.1) package “vegan” (version 2.4-3). Partial least squares discriminant analysis (PLS-DA) was performed by using R (version 3.3.1) package “mixOmics” to discriminate the community structure in different groups. Heatmaps, ternary plots, and circus plots were conducted in R (version 3.3.1) package “vegan” (version 2.4-3), ggtern plug-in, and Circos software (version 0.67-7), respectively. The relationship between microbial community and environmental factors was analyzed by redundancy analysis (RDA) using vegan package. In typing analysis, samples were clustered into five types with the highest Calinski–Harabasz (CH) index determined by the partitioning around medoids (PAM) algorithm. Linear discriminant analysis (LDA) of effect size (LEfSe) was executed to identify the significant taxa that most likely explained the differences between groups, with a threshold LDA score of 2 or greater used. For correlation analysis, Spearman’s rank test was performed and results were visualized in heatmap. A *p*-value of <0.05 was considered statistically significant in the current study.

## Results

### Overview of the Subjects and Samples

Fifty-five saliva samples were collected from 15 children with ECC, 22 children with SECC, and 18 CF controls, with a mean ± SD age of 45.1 ± 9.6 months. **Table 1** and **Table S1** show some characteristics of the study participants. As for the bacterial community, a total of 2,941,799 16S rRNA reads were obtained, with an average of 53,487 per sample. After rarefying the OTU table to the minimum number of reads (20,398) per sample, a total of 13,183 OTUs were identified and were assigned to 535 species and 201 genera. As for the fungal community, a total of 4,169,098 ITS reads were obtained, with an average of 75,801 per sample. After rarefying the OTU table to the minimum number of reads (19,686) per sample, a total of 15,023 OTUs were identified and were assigned to 784 species and 445 genera.

**Table 1:** Demographics and carious characteristics of the study participants.

Variable	Caries severity		
	CF (*n* = 18)	ECC (*n* = 15)	SECC (*n* = 22)
Age (months)	40.2 ± 6.9	46.9 ± 9.2	45.1 ± 10.5
Gender (M/F)	13/5	5/10	9/13
dmfs score	0	2 ± 0.8	10.5 ± 8.1
dmft score	0	1.9 ± 0.2	6.4 ± 3.3

### Salivary Bacterial Profile in Children With ECC

**Figure 1:**
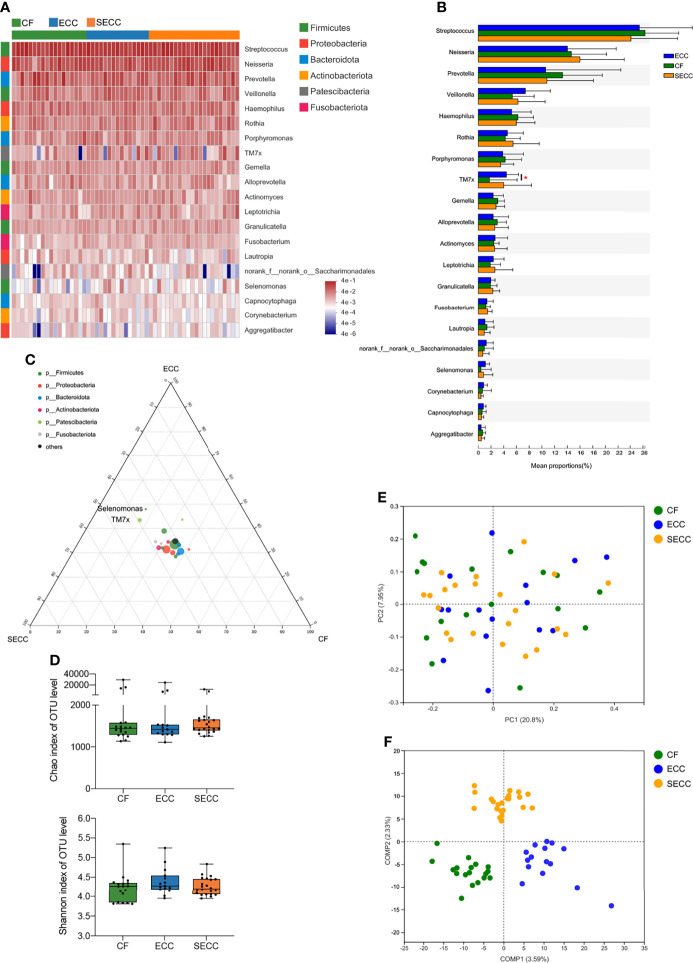
Salivary bacterial profile in children with different health conditions. **(A)** Overview of the 20 most abundant salivary genera, and the heatmap plot presents the common logarithm of relative abundance. **(B)** Statistical comparison of the relative abundance of the 20 most abundant genera (Kruskal–Wallis, among three groups; Wilcoxon rank-sum test, within two groups; *p < 0.05). **(C)** The relative occurrence of genera (circles) in samples with different oral health conditions. **(D)** Alpha-diversity indices for richness (Chao index, Kruskal–Wallis, *p* = 0.351) and diversity (Shannon index, Kruskal–Wallis, *p* = 0.351) of the bacterial community on the OTU level. **(E)** Principal coordinates analysis (PCoA) plots of Bray–Curtis dissimilarities at the OTU level (PERMANOVA, *p* = 0.771). **(F)** Partial least square discriminant analysis (PLS-DA) plot of saliva microbiota. CF, caries free; ECC, early childhood caries; OTU, operational taxonomic unit; PCoA, principal coordinates analysis; PERMANOVA, permutational multivariate analysis of variance; PLS-DA, partial least square discriminant analysis; SECC, severe early childhood caries.

Salivary bacterial community of the current cohort mainly consisted of six phyla, namely, *Firmicutes*, *Proteobacteria*, *Bacteroidota*, *Actinobacteriota*, *Patescibacteria*, and *Fusobacteriota*. *Streptococcus* (CF: 26.32%; ECC: 25.44%; SECC: 24.12%) was the most abundant genus in the saliva sample, followed by *Neisseria* (CF: 14.63%; ECC: 14.02%; SECC: 16%), *Prevotella* (CF: 13.24%; ECC: 10.59%; SECC: 10.79%), *Veillonella* (CF: 5.349%; ECC: 7.402%; SECC: 6.243%), and *Haemophilus* (CF: 6.229%; ECC: 5.245%; SECC: 5.98%). The taxonomic profile of the top 20 most abundant bacterial genera is shown in **Figure 1A**, with mean ± SD relative abundances in each group listed in **Table S2**. Although no significant differences in the relative abundance of the top 20 bacterial genera were observed among three groups (Kruskal–Wallis; **Figure 1B**), *TM7x* in ECC children presented a significantly higher level than that in CF children (Wilcoxon rank-sum test, *p* = 0.04879). The *p*-values of difference statistics of the top 20 bacterial genera are listed in **Table S2**. As for taxa whose relative abundance was less than 1%, *Centipeda*, *Parvimonas*, and *Pseudopropionibacterium* also showed different relative abundance among three groups or between two groups, and *p*-values of difference statistics are listed in **Table S3**. We then employed ternary analysis to evaluate the taxa distribution corresponding to different oral health conditions, and a quite narrow distribution of bacterial genera was observed (**Figure 1C**). *TM7x* and *Selenomonas*, whose relative abundance was more than 1% in at least one group, were found enriched in caries samples. No significant difference in richness (Chao index, Kruskal–Wallis, *p* = 0.351; **Figure 1D**), alpha-diversity (Shannon index, Kruskal–Wallis, *p* = 0.351; **Figure 1D**), or beta-diversity (Bray–Curtis, PERMANOVA, *p* = 0.771; **Figure 1E**) existed among the CF, ECC, and SECC groups. ANOSIM also indicated no significant difference of bacterial structure within groups (**Table 2**). However, PLS-DA suggested an obviously different structure of overall saliva bacteria among three groups (**Figure 1F**).

**Table 2:** The calculations and *p*-values of ANOSIM and Adonis of salivary bacteria and fungi.

		Bray–Curtis ANOSIM		Adonis	
		Statistic	*p*-value	*r* ^2^	*p*-value
Bacteria	Among three groups	−0.0045	0.522	0.0309	0.764
	CF vs. ECC	0.0136	0.291	0.0322	0.362
	ECC vs. SECC	−0.0383	0.779	0.0161	0.975
	CF vs. SECC	0.0097	0.299	0.0228	0.555
Fungi	Among three groups	0.0477	0.052	0.0393	0.283
	CF vs. ECC	−0.0193	0.634	0.0261	0.742
	ECC vs. SECC	0.0945	0.05	0.0331	0.136
	CF vs. SECC	0.0576	0.046	0.0294	0.174

CF, caries free; ECC, early childhood caries; SECC, severe early childhood caries.

### Salivary Fungal Profile in Children With ECC

**Figure 2:**
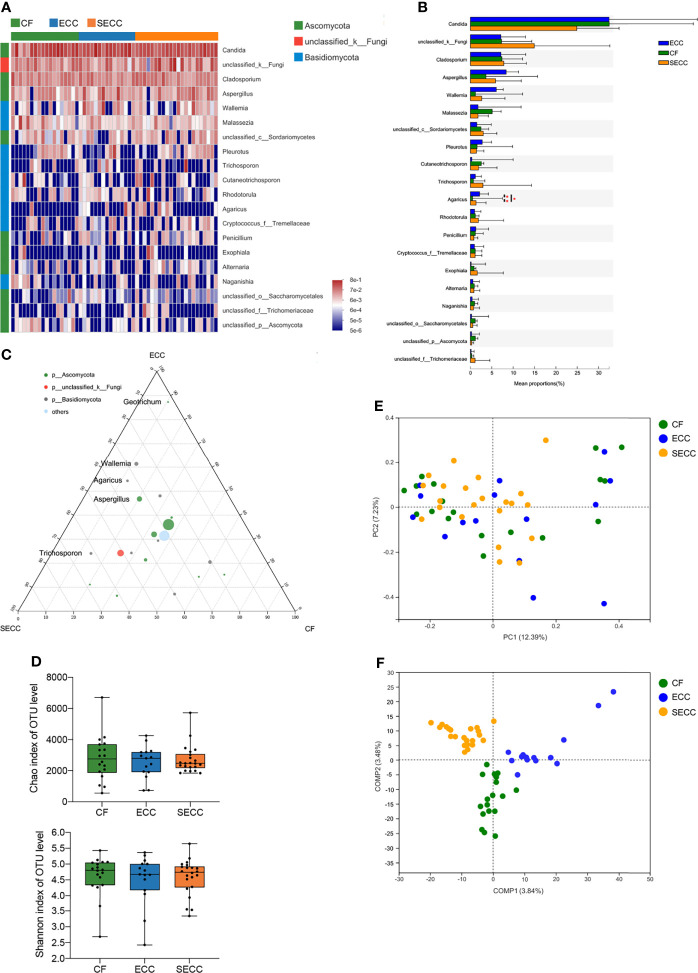
Salivary fungal profile in children with different health conditions. **(A)** Overview of the 20 most abundant salivary genera, and the heatmap presents the common logarithm of relative abundance. **(B)** Statistical comparison of the relative abundance of the 20 most abundant genera (Kruskal–Wallis, among three groups; Wilcoxon rank-sum test, within two groups; *p < 0.05). **(C)** The relative occurrence of genera (circles) in samples with different oral health conditions. **(D)** Alpha-diversity indices for richness (Chao index, Kruskal–Wallis, *p* = 0.886) and diversity (Shannon index, Kruskal–Wallis, *p* = 0.823) of the fungal community on the OTU level. **(E)** PCoA plots of Bray–Curtis dissimilarities at the OTU level (PERMANOVA, *p* = 0.304). **(F)** PLS-DA plot of saliva microbiota. CF, caries free; ECC, early childhood caries; OTU, operational taxonomic unit; PCoA, principal coordinates analysis; PERMANOVA, permutational multivariate analysis of variance; PLS-DA, partial least square discriminant analysis; SECC, severe early childhood caries.

*Ascomycota* and *Basidiomycota* constituted the majority of salivary fungal community at the phylum level. *Candida* (CF: 32.49%; ECC: 32.45%; SECC: 24.91%) was the most abundant fungal genus, and *Cladosporium* (CF: 7.368%; ECC: 7.156%; SECC: 7.81%), *Aspergillus* (CF: 3.638%; ECC: 8.364%; SECC: 5.873%), *Wallemia* (CF: 1.178%; ECC: 6.06%; SECC: 2.634%), and *Malassezia* (CF: 5.088%; ECC: 1.772%; SECC: 1.773%) were also enriched in the saliva samples. The taxonomic profile of the top 20 most abundant fungal taxa is shown in **Figure 2A**, and mean ± SD relative abundances in each group are listed in **Table S4**. In the top 20 fungal taxa, *Agaricus* showed significantly different levels among three groups (Kruskal–Wallis, *p* = 0.04708), presenting higher relative abundance in either ECC or SECC group than that in the CF group (Wilcoxon rank-sum test, *p* = 0.02608 and *p* = 0.02014, respectively) (**Figure 2B**). The *p*-values of difference statistics are listed in **Table S4**. As for fungal taxa whose relative abundance was less than 1%, *unclassified_o:Hypocreales*, *Bjerkandera*, *Mycosphaerella*, *Subulicystidium*, *Neoascochyta*, and *Psathyrella* also showed significant difference in relative abundance among three groups or between healthy and caries samples, and *p*-values of difference statistics are listed in **Table S5**. Ternary analysis revealed a dispersed distribution of fungal genera, indicating that certain taxa might be relevant to healthy or caries status (**Figure 2C**). *Aspergillus*, *Wallemia*, *Agaricus*, and *Geotrichum*, whose mean relative abundances were more than 1%, were found enriched in carries samples. Beyond our expectation, there was no obvious difference in *Candida* level among healthy and caries samples, indicating the potential caries-promoting effect of fungal genera other than *Candida*. Although no significant difference in richness (Chao index, Kruskal–Wallis, *p* = 0.886; **Figure 2D**), alpha-diversity (Shannon index, Kruskal–Wallis, *p* = 0.823; **Figure 2D**), or beta-diversity (Bray–Curtis, PERMANOVA, *p* = 0.304; **Figure 2E**) was observed within three groups, ANOSIM revealed remarkable variations of salivary fungal structure between SECC and other two groups (**Table 2**). PLS-DA also indicated a distinct fungal structure among three groups (**Figure 2F**).

### Association Between Saliva Fungal Types and Oral Health Status

**Figure 3:**
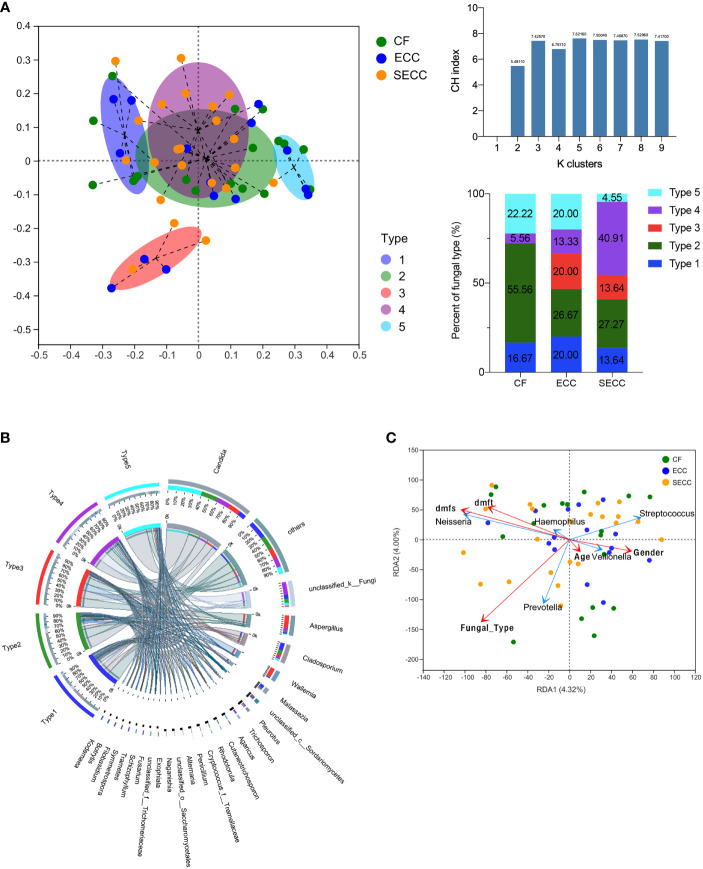
Association between salivary fungi and oral health condition and salivary bacterial profile. **(A)** The distribution of samples based on the highest CH indices using the PAM algorithm (Bray–Curtis distance) and the proportions of five fungal types in three groups. **(B)** Profiles of each fungal type at the genus level. **(C)** Correlation between salivary bacterial community structure and host factors by using RDA. CF, caries free; CH indices, Calinski–Harabasz indices; ECC, early childhood caries; PAM, partitioning around medoids; RDA, redundancy analysis; SECC, severe early childhood caries.

Since insignificant bacterial variations but notable fungal alterations were detected within healthy and caries samples, we intended to further focus on the association between saliva fungi and oral health status. By executing PAM and PCoA in terms of Bray–Curtis distance, the fungal community of all samples was clustered into five types at the genus level (**Figure 3A**). Typing analysis revealed that 55.56% (10/18) CF samples owned the type 2 fungal community, while this fungal type occurred in only 26.67% (4/15) ECC samples and 27.27% (6/22) SECC samples. No CF sample possessed the type 3 fungal community, which was found in 20.00% (3/15) ECC samples and 13.64% (3/22) SECC samples. The type 4 fungal community was detected in 13.33% (2/15) ECC samples and up to 40.91% (9/22) SECC samples, but was found in only one CF sample. Type 1 and type 5 fungal communities were evenly distributed in the healthy and caries cases. The distinctive composition and structure indicated the uniqueness of the five fungal types (Bray–Curtis, PERMANOVA, *p* = 0.001; **Figure 3B**), even though type 3 and type 4 were both clustered from caries-related samples. To reflect the sample condition that each fungal type prevailed over, we designated type 2 as health-related type (H type), type 3 and type 4 as caries-related type 1 (C-1 type) and caries-related type 2 (C-2 type), and type 1 and type 5 as general type 1 (G-1 type) and general type 2 (G-2 type). After eliminating *unclassified_k:Fungi*, we found that five fungal types were dominated by different genera: *Candida* followed by *Cladosporium* and *Cutaneotrichosporon* (H type), *Aspergillus* followed by *Candida* and *Wallemia* (C-1 type), *Candida* followed by *Aspergillus* and *Trichosporon* (C-2 type), *Cladosporium* followed by *Candida* and *Malassezia* (G-1 type), and *Candida* followed by *Cladosporium* and *Agaricus* (G-2 type), and other less abundant genera were also distinctive in different types (**Table S6**). Since all five types possessed a relatively high abundance of *Candida*, we came up with the hypothesis that it might be the fungal taxa other than *Candida* that led to the different oral health conditions in the current cohort. The typing analysis was also executed on the bacterial community at the genus level in terms of Bray–Curtis distance, with no obvious association between bacterial types and oral health status being observed (**Figure S1** and **Table S7**).

Due to the pathogenetic roles of certain bacteria in ECC onset, we intended to investigate whether saliva fungi can impact bacterial profile. The fungal type corresponding to each sample was considered as an affecting factor, and host factors including age, gender, dmfs score, dmft score, and fungal type were assessed regarding their influence on the bacterial community by performing RDA. RDA revealed that fungal type was the only factor that significantly affected the bacterial community structure (*r* ^2^ = 0.1118, *p* = 0.044), with its close relationship to the distribution of *Prevotella*, a potential bacterial biomarker of ECC (**Figure 3C** and **Table 3**). Thus, we came up with the opinion that the oral fungal community can influence bacterial profiles, and this cross-kingdom interaction may result in different oral health status.

**Table 3:** The calculations and *p*-values of redundancy analysis.

	RDA1	RDA2	*r* ^2^	*p*-values
Age	0.7267	−0.6869	0.0015	0.964
Gender	0.9913	−0.1319	0.0239	0.547
dmfs	−0.9789	0.2044	0.0788	0.099
dmft	−0.9543	0.2989	0.0488	0.254
Fungal_Type	−0.7945	−0.6072	0.1118	0.044

### Potential Crosstalk Between the Oral Microbiome and Mycobiome in Children With ECC

**Figure 4:**
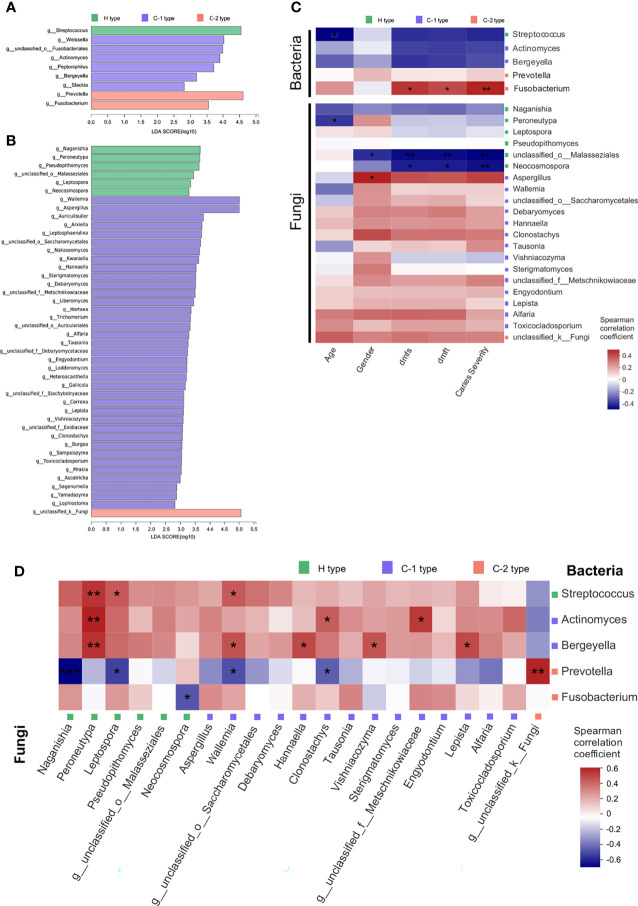
Potential crosstalk between the oral microbiome and mycobiome in children with different oral health status. **(A)** LDA scores of distinct salivary bacteria among groups at the genus level. **(B)** LDA scores of distinct salivary fungi among groups at the genus level. **(C)** Spearman rank correlation between host properties and abundances of distinct salivary bacteria and fungi. **(D)** Spearman rank correlation between distinct salivary bacteria and fungi. *p < 0.05, ** < 0.01, ***p < 0.001. C-1 type, caries-related type 1; C-2 type, caries-related type 2; dmfs, decayed, missing, or filled surfaces; dmft, decayed, missing, or filled teeth; H type, health-related type; LDA, linear discriminant analysis.

Since we detected the possible relationship between the oral fungal and bacterial community, we intended to identify the specialized taxa that distinguished samples from healthy to caries conditions. Samples were divided into five groups as previously clustered by typing analysis. For a better consistency within the actual health state and dominant fungal type of each cluster, we removed the CF sample in the caries-related cluster, as well as ECC or SECC samples in the health-related cluster. We then used LEfSe to identify the taxa that significantly differed between health and caries samples, and bacterial or fungal genera with LDA scores of 2 or greater were confirmed and shown in **Figures 4A, B**, respectively. As for the bacterial community, *Streptococcus* was enriched in the health-related type community, while *Actinomyces*, *Prevotella*, and *Fusobacteria* were enriched in the caries-related type community. As for the fungal community, a wealth of taxa was found assembling in caries-related type community. Focusing on the prevailing taxa, we eliminated the genera whose relative abundances were less than 1%, remaining 5 bacterial genera and 21 fungal genera. The correlation between filtered taxa and host properties was then determined by using Spearman correlation analysis. It was found that the bacterial taxon *Fusobacterium* was positively related to dmfs score, dmft score, and caries severity, while the negative correlations between either *unclassified_o:Malasseziales* or *Neocosmospora* and caries phenotypes were figured out (**Figure 4C** and **Table S8**). Thus, we deduced that *Fusobacterium* plays an important role in caries onset, whereas *unclassified_o:Malasseziales* and *Neocosmospora* may represent good oral health condition. The relationship between filtered bacterial and fungal taxa was further assessed, revealing 17 significant correlation in cross-kingdom pairs (**Figure 4D** and **Table S9**). As for health-related taxa, fungal taxa *Leptospora* was positively related to bacterial taxa *Streptococcus* (*p* = 0.042), while negative correlations between *Naganishia* and *Leptospora* and caries-related *Prevotella* (*p* = 0.00044; *p* = 0.011) were observed. Of note, the significant negative correlation between *Neocosmospora* enriched in CF samples and *Fusobacterium* on behalf of caries was revealed (*p* = 0.024), reconfirming the opposite roles they played in different oral health conditions. As for caries-related taxa, fungal taxa including *Hannaella*, *Vishniacozyma*, *unclassified_f:Metschnikowiaceae*, and *Lepista* were found positively related to bacterial taxa *Actinomyces* or *Bergeyella* (*p* = 0.023; *p* = 0.035; *p* = 0.012; *p* = 0.022).

## Discussion

In the current study, we investigated the salivary bacterial and fungal profiles in children with good oral health, ECC, and SECC. Most studies involving ECC were largely focused on oral bacteria or pathogenic *C. albicans* (Xiao et al., 2018; Alkhars et al., 2021; Baker et al., 2021; de Jesus et al., 2021). Here, we noticed the novel correlation between integral oral mycobiome and microbiome, figuring out potential taxonomic biomarkers which may lead to different oral health conditions in children. The fungal community was found distinguished into five types on behalf of different oral health status and significantly affected the bacterial profile. By analyzing the correlation between enriched taxa and carious indexes, we found *Neocosmospora* and *Fusobacterium* could be considered as potential biomarkers of good oral health and caries risk, respectively. Therefore, we concluded that the joint effect of salivary fungi and bacteria may play important roles in caries progression, and the impact of oral mycobiome on oral health deserved further investigation.

ECC is the most common disease of children with high prevalence worldwide, presenting as one or more decayed, missing, or filled tooth/surfaces in the primary tooth (Casamassimo et al., 2009; Anil and Anand, 2017). It has been substantiated that the co-infection of *S. mutans* and *C. albicans*, accompanied by bad oral hygiene, genetic factor, and immunological factor, led to the onset of ECC (Xiao et al., 2018) and, meanwhile, resulted in the dysbiosis of the oral microbiome (Kressirer et al., 2018; Xiao et al., 2018; Baker et al., 2021). Numerous studies have investigated the changes of the oral bacterial community in caries children compared with CF children, detecting decreased bacterial diversity as well as identifying certain discriminatory taxa including *Streptococcus*, *Prevotella*, *Veillonella*, *Neisseria*, and *Rothia* that were associated with caries (Wang et al., 2019; de Jesus et al., 2020; Baker et al., 2021; de Jesus et al., 2021). To our surprise, no significant difference of bacterial alpha- or beta-diversity based on caries status existed in the present cohort, although the non-distinctive alpha-diversity was also reported by Grier et al. and Agnello et al. (Agnello et al., 2017; Grier et al., 2021). Among the bacteria genera with high relative abundance, only *TM7x* was found enriched in children with ECC, whose strong co-occurrence with caries risk has been previously noticed (Kalpana et al., 2020; Baker et al., 2021).

In recent years, researchers have gradually attached more attention to the relationship between oral fungi and caries development. Most ITS-based investigations were focused on the dental plaque, revealing an increased fungal load, decreased community diversity, and enrichment of several taxa including *C. albicans*, *Candida dubliniensis*, *Candida sake*, *Cryptococcus neoformans*, and *Nigrospora oryzae* in samples from subjects with caries, while *Malassezia globose*, *Bipolaris sorokiniana*, *Mycosphaerella*, and *Trichosporon* were more relevant to CF status (Baraniya et al., 2020; de Jesus et al., 2020; O'Connell et al., 2020; Cui et al., 2021). *In vitro*, despite the well-recognized synergistic interaction between *S. mutans* and *C. albicans*, *M. globose* enriched in CF subjects has demonstrated its inhibitory properties against *S. mutans* (Baraniya et al., 2020). In the present study, we observed high relative abundances of *Candida*, *Cladosporium*, *Aspergillus*, and *Malassezia* across subjects with or without caries, which have been consistently identified as core human oral mycobiome in previous studies (Ghannoum et al., 2010; Fechney et al., 2019; Baraniya et al., 2020). Significant differences of beta-diversity and distribution of fungal colonies were noticed, indicating a higher colony sensitivity toward oral health conditions than that of the bacterial community. The obvious enrichment of *Candida* did not exist in caries samples, and previous studies also failed to detect the increased abundance of *C. albicans* in children with caries (Fechney et al., 2019; de Jesus et al., 2020), which suggests the potential pathogenic impact of other fungi in ECC development. The fungal community was determined into five discriminative fungal types *via* typing analysis, and types were designated as general type, health-related type, and caries-related type according to the prevalence in CF or caries samples. Previous studies have observed that certain *Candida* species can influence caries risk by affecting oral bacteria profile (Xiao et al., 2018; de Jesus et al., 2020; Alkhars et al., 2021). By performing RDA, we also verified the significant impact of fungal types on bacterial profile. RDA further revealed the positive correlation between fungal types and the distribution of *Prevotella*, a critical biomarker of ECC onset with great contribution to acid production (Yang et al., 2012; Teng et al., 2015; Wang et al., 2019; Baker et al., 2021). Surprisingly, although *Neisseria* has long been found associated with good oral health (Baker et al., 2021; Qudeimat et al., 2021), the strong positive correlation between *Neisseria* and either dmfs or dmft score was observed in the present study, indicating its potential cariogenic capabilities such as sugar metabolism, acid production, and acid tolerance in caries risk (Xiao et al., 2021).

Typing analysis also clustered saliva samples into five groups, three of which were dominated by CF or caries samples, being termed as health-related cluster or caries-related clusters. In order to precisely lock on the symbolic taxa of healthy or caries condition, we firstly filtered samples by eliminating those whose actual health status was not concordant to the cluster type. The characteristic genera of either health-related cluster or caries-related clusters were then identified by executing LEfSe, and taxa with relative abundance above 1% were retained for further evaluation. The relationship between filtered taxa and host properties was assessed by Spearman correlation analysis. Being the member of the “orange” complex in subgingival plaque, *Fusobacterium* was traditionally regarded as a critical periodontal pathogen (Socransky et al., 1998), while increasing studies have noticed its enrichment and predictive potentiality on ECC occurrence (Zhu et al., 2018; Chen et al., 2021). Here, a significant positive correlation between *Fusobacterium* and caries indexes was observed, re-emphasizing its possible roles in caries progression. On the contrary, *unclassified_o:Malasseziales* and *Neocosmospora* were identified as negatively correlated to caries indexes. *Malassezia* is a commonly detected fungus of human skin and oral cavity (Gaitanis et al., 2012; Baraniya et al., 2020) and was previously found significantly enriched in CF children (Baraniya et al., 2020). *Neocosmospora* is an originally reported oral health-relevant fungus in current research, which includes groups of taxa that were previously assigned to the *Fusarium solani* complex discovered from plants, humans, and animals (Sandoval-Denis et al., 2018). It was revealed that galactose oxidase secreted by *Fusarium* species can convert substrates into the aldehyde forms and concomitantly produce hydrogen peroxide, and its ability in inhibiting *S. mutans* has been demonstrated (Lis and Kuramitsu, 1997). Further Spearman correlation analysis substantiated the significant negative correlation between *Fusobacterium* and *Neocosmospora*, indicating their opposite effects in maintaining CF condition or developing caries. In addition, although a number of studies have verified the promoting effects of *S. mutans* in ECC progression (Momeni et al., 2020; Xiao et al., 2020; Baker et al., 2021), *Streptococcus* was found to be a characteristic in the CF sample and, meanwhile, negatively related to carious indexes in the present study, indicating the complicated roles that the *Streptococcus* species played in caries risk, and the intricate prevalence of *Streptococcus* species has also been reported by AlEraky et al. (2021).

Caution should be taken when applying current evidence. Firstly, community analysis was restrained at the genus level due to the shallow sequencing depth, which provided a less-precise profile of community variation and was unable to lock on the exact species or subspecies that are closely related to ECC risk. On account of the small sample size, we did not conduct random forest model with receiving operational curve (ROC) analysis to test the discriminatory power of genera signature, leaving a less demonstration quality of the results. The limitations above remind us of a larger-scale cohort and the employment of whole metagenome sequencing and metabolomics analysis in future studies. Although saliva samples are easy to collect, the distinctive taxonomic profiles between saliva and dental plaque have been observed in previous research (Cui et al., 2021; de Jesus et al., 2021), suggesting a further investigation of plaque microorganisms in predicting ECC, for the closer contact of plaque with the dental surface may result in a higher sensitivity in predicting ECC compared with that of saliva samples. Besides, attention need to be paid to the cross-kingdom interactions in ECC progression, and the mechanisms of *Neocosmospora* in inhibiting cariogenic taxa are worthy of future investigation.

## Data Availability Statement

The datasets presented in this study can be found in online repositories. The names of the repository and accession numbers can be found below: NCBI; accession numbers: PRJNA790078 and PRJNA790007.

## Ethics Statement

The studies involving human participants were reviewed and approved by the Institutional Review Board of West China Hospital of Stomatology. Written informed consent to participate in this study was provided by the legal guardian/next of kin of the participants. Written informed consent was obtained from the legal guardian/next of kin of the minor(s) for the publication of any potentially identifiable images or data included in this article.

## Author Contributions

YZ and XZ contributed to the study design and critically revised the manuscript. YT contributed to the conception, bioinformatic analysis, data interpretation, and manuscript drafting. ZZ and CS contributed to the sample collection, data acquisition, and data analysis. All authors contributed to the article and approved the submitted version.

## Funding

This study was supported by the National Natural Science Foundation of China (81870754, 81700964).

## Conflict of Interest

The authors declare that the research was conducted in the absence of any commercial or financial relationships that could be construed as a potential conflict of interest.

## Publisher’s Note

All claims expressed in this article are solely those of the authors and do not necessarily represent those of their affiliated organizations, or those of the publisher, the editors and the reviewers. Any product that may be evaluated in this article, or claim that may be made by its manufacturer, is not guaranteed or endorsed by the publisher.
