# Supplementary material for: The Crosstalk Between Saliva Bacteria and Fungi in Early Childhood Caries
Source: Front Cell Infect Microbiol. 2022 Feb 14;12:845738. doi: 10.3389/fcimb.2022.845738 (PMC8884336; doi:10.3389/fcimb.2022.845738)
Supplement: Supplementary file 1 [file DataSheet_1.docx]

Supplementary Material

## Supplementary Figures


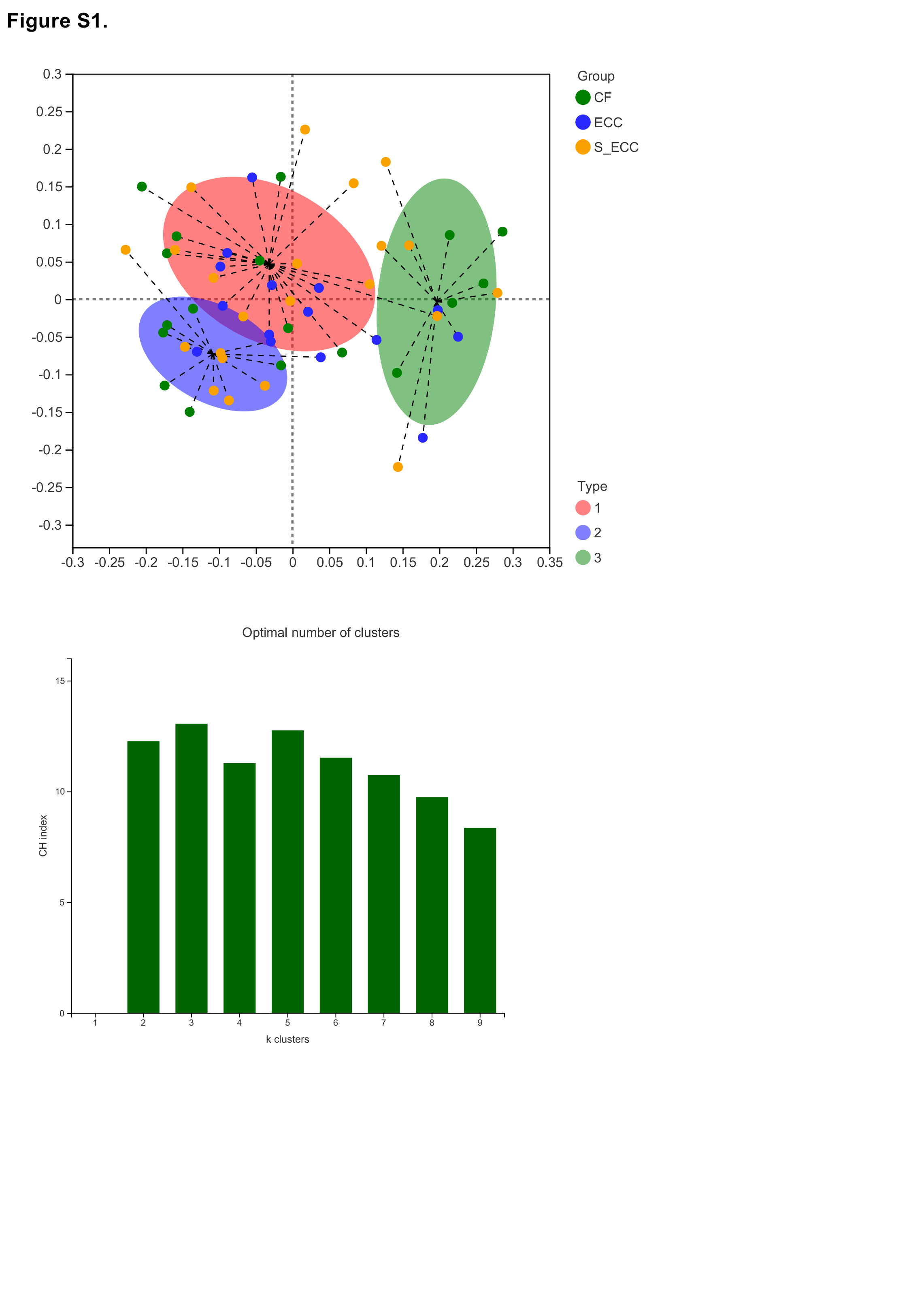


**Figure S1.** Typing analysis of salivary bacterial community. The distribution of samples based on the d highest CH indices using the PAM algorithm (Bray-Curtis distance). CH indice, Calinski-Harabasz indices; PAM, Partitioning Around Medoids.
